# CD19/CD22 bispecific CAR-T cells for MRD-positive adult B cell acute lymphoblastic leukemia: a phase I clinical study

**DOI:** 10.1038/s41408-023-00813-x

**Published:** 2023-03-24

**Authors:** Jiahua Niu, Huiying Qiu, Fang Xiang, Lin Zhu, Jun Yang, Chongmei Huang, Kun Zhou, Yin Tong, Yu Cai, Baoxia Dong, Yuan Lu, Xuedong Sun, Liping Wan, Xueying Ding, Haopeng Wang, Xianmin Song

**Affiliations:** 1 Department of Hematology, Shanghai General Hospital, Shanghai Jiaotong University School of Medicine, Shanghai, China; 2 Engineering Technology Research Center of Cell Therapy and Clinical Translation, Shanghai Science and Technology Committee (STCSM), Shanghai, China; 3 Hrain Biotechnology, Shanghai, China; 4 School of Life Science and Technology, Shanghai Tech University, Shanghai, China

**Keywords:** Phase I trials, T cells, Acute lymphocytic leukaemia

**Dear Editor**,

Measurable residual disease (MRD) is now considered as one of the most important prognostic factors for B cell acute lymphoblastic leukemia (B-ALL), even for patients undergoing allogeneic hematopoietic stem cell transplantation (allo-HSCT) [1, 2]. Early achievement of MRD negative is imperative, as many studies consistently demonstrated that the patients with a negative MRD during the early time of treatment, especially after induction, had a superior survival [3]. Chimeric antigen receptor T (CAR-T) cells targeting CD19 or CD22 has been demonstrated as the most effective salvage therapy for refractory/relapsed B-ALL [4, 5]. Considering the facts that a lower disease burden was associated with a higher safety profile and dual targeting of CD19/CD22 might lower relapse risk, we designed a phase I study to evaluate the safety and efficacy of CD19/CD22 bispecific targeted CAR-T cells for MRD-positive adult B-ALL patients, especially for primary patients with MRD persistence after early consolidation therapy.

Adult MRD positive B-ALL patients were enrolled into the clinical trial (NCT: 03919526). Primary patients with MRD persistence after induction and at least 2 courses of consolidation therapy were defined as first-line consolidation group; while recurrent patients with MRD-positive complete remission (CR) or CR with incomplete hematological recovery (CRi) after salvage therapy or patients with MRD relapse were defined as relapsed group. The details of inclusion/exclusion criteria were shown in Supplementary Table 1.

The study was an open, phase I clinical trial with a sample size of 16–18. The primary objective was to assess the safety of CD19/CD22 bispecific CAR-T cells for MRD positive B-ALL, and the secondary was to evaluate its efficacy. We performed traditional 1 + 1 + 3 + 3 dose escalation to determine the optimal single dose of CAR-T cells, which corresponded to four dose levels of 1 × 10^6^ cells/kg, 2 × 10^6^ cells/kg, 3 × 10^6^ cells/kg, and 5 × 10^6^ cells/kg, respectively. The study design was summarized in supplementary materials,methods, and Supplementary Fig. 1.

Fifteen out of 19 patients completed CAR-T cell infusion from March 2019 to May 2022. Four did not due to disease progression (2/4) and complete MRD response to graft versus host disease (2/4). The baseline characteristics of the 15 patients were summarized in Table 1 and Supplementary Table 2. Transduction efficiency and infused doses of CAR-T cells were shown in the Supplementary table 3. The adverse events (AEs) within 28 days after infusion were shown in Supplementary table 4. All the 15 patients experienced grade 3 or higher AEs and the most common were cytopenia. A total of 4 patients (26.7%, 4/15) developed grade 1 or 2 cytokine-release syndrome (CRS) and all were in 5 × 10^6^/kg CAR-T cell dose group. Two patients (NO. 007 and NO. 015) with grade 2 CRS were treated with tocilizumab and recovered quickly. The median time of CRS onset was 2 (range, 1–8) days after infusion, and the median duration was 1 (range, 1–3) day. One patient (No.009) developed delayed- neurotoxicity on day 230 after CAR-T infusion and recovered quickly with prednisone (15 mg/d) (Details were described in supplementary materials).

**Table 1:** Patient characteristics.

Baseline characteristics	All patients evaluable (*N* = 15)	First-line consolidation (*N* = 11)	Relapsed group (*N* = 4)	*P*
Median age, years (range)	51 (23–70)	45 (23–70)	54.5 (31–61)	0.447
Age ≥ 35 years, *n* (%)	10(66.7%)	7(63.6%)	3(75%)	0.68
Male, *n* (%)	6 (40%)	5 (45.5%)	1 (25%)	0.348
ECOG performance status score of 0–1, *n* (%)	15 (100%)	11 (100%)	4 (100%)	>0.99
Median time since diagnosis, months (range)	6.5 (1–41.5)	6.5 (3–18)	7 (1–41.5)	0.851
Median Cycle Number of chemotherapy, *n* (range)	4(3–8)	4(3–5)	3.5 (3–8)	0.949
Ph-positive	7(46.7%)	3(27.3%)	4(100%)	0.026
Disease burden				
Before lymphodepletion				
MRD ≥ 10^–2^	2(13.3%)	1(9.1%)	1(25%)	0.4
MRD ≥ 10^–3^–<10^–2^	5(33.3%)	3(27.3%)	2(50%)	
MRD ≥ 10^–4^–<10^–3^	8(53.3%)	7(63.6%)	1(25%)	
Before infusion				
MRD ≥ 10^–2^	3(21.4%)	1(9.1%)	2(50%)	0.183
MRD ≥ 10^–3^–<10^–2^	5(35.7%)	5(45.5%)	0	
MRD ≥ 10^–4^–<10^–3^	1(7.1%)	1(9.1%)	0	
MRD Negative	5(35.7%)	3(27.3%)	2(50%)	
Follow-up time	15.5(2.5–33)	15.5(2.5–33)	15.25(10.5–20.5)	0.949

As shown in Fig. 1, the median follow-up time was 15.5 months (range, 2.5–33). Five patients achieved MRD-negative CR before infusion (after lymphodepletion). The overall MRD response rate was 100% at day 28 with 93.9% (14/15) MRD-negative CR and 6.7% (1/15) MRD-negative CRi. Eleven remained MRD-negative until the end of the follow-up. Five patients experienced morphological or MRD relapse (3 in the first-line consolidation group and 2 in the relapsed group) at follow-up (Details in supplementary materials).

**Fig. 1:**
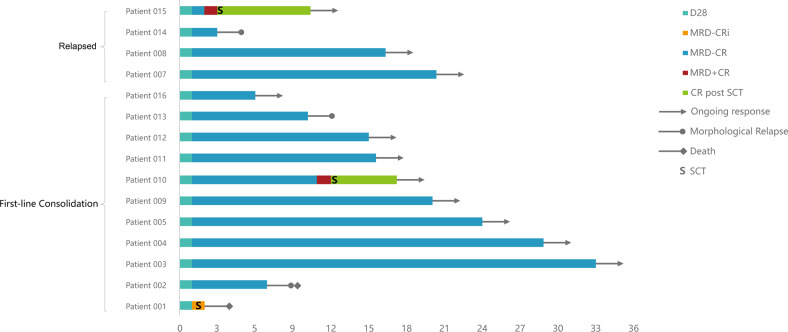
Clinical outcomes of patients with MRD-positive B-ALL after CD19/CD22 bispecific CAR-T cells. The bar chart shows the clinical response and follow-up of patients during CAR-T therapy. Each bar represents an individual patient and the study number. The different colors represent different disease status.

For total patients, the median relapse free survival (RFS) and overall survival (OS) were not reached (Supplemental Fig. 2A, B). The 12-month RFS and OS were 77% (95% CI, 55–99) and 86% (95% CI, 68–104), respectively, while the 24-month estimated RFS and OS were the same as those of the 12-month. Median RFS and OS were similar between patients with/without subsequent transplantation after CAR-T therapy (*P* = 0.735 for RFS, *P* = 0.671 for OS) (Supplementary Fig. 2A–B) and patients in the first-line consolidation /relapsed groups (*P* = 0.803 for RFS, *P* = 0.369 for OS) (Supplementary Fig. 3). Neither MRD status before CAR-T cells nor Ph-positive/negative affected the survival (Supplementary Fig. 4A–D).

For the 11 patients in the first-line consolidation group, the median RFS and OS were not reached (Supplementary Fig. 2C, D), the 12-month RFS and OS were 77.8% (95% CI, 51–105) and 80.8% (95% CI, 57–105). Except for one patient with subsequent transplantation after CAR-T cells, the 10 patients had a 12-month RFS of 77.8% (95% CI, 51–105) and OS of 88.9% (95% CI, 68–109). The 24-month estimated RFS and OS for all patients and patients except one with subsequent transplantation were the same as those of 12 months. Five out of the 11 patients had a RFS of more than 18 months (Fig. 1). Stratified survival analysis showed no significant differences in RFS and OS between patients with Ph-positive/negative B-ALL (*P* = 0.432 for RFS, *P* = 0.417 for OS), and different MRD status before infusion (*P* = 0.379 for RFS, *P* = 0.593 for OS) (Supplementary Fig. 5A–D).

Peripheral blood CAR T-cell expansion was observed in all 15 patients, with a median time to reach the peak CAR-T cell concentration (Cmax) of 10 days (range, 7–14). Cmax and AUC_0–28_ of CAR T-cell expansion were higher in patients with sustained remission than that with relapse (*P* = 0.048 for Cmax and *P* = 0.018 for AUC_0–28d_, respectively). CAR-T cell persistence in peripheral blood with >100 copies/μg DNA lasted for more than 60 and 90 days in 7 and 3 patients, respectively and decreased significantly in the rest patients within 28 days after infusion. The details of pharmacokinetics and pharmacodynamics were shown in supplementary materials and Supplementary Fig. 6A, B. Cytokines were routinely detected after CAR-T cell infusion (Supplementary Table 5). The elevated levels of cytokines (CRP, IL-6, etc.) had no significant differences between patients with and without CRS, as well as between patients with relapse and sustained remission (*P* > 0.05) (Supplementary Fig. 7A–F). The lymphocyte subtype numbers of all patients at different times pre- and post-infusion were shown in Supplementary Fig. 8 and Supplementary Table 6.

In the present study, only 26.7% of patients developed CRS without severe CRS and early neurotoxic effects, although a high dose of CD19/22 CAR-T cells of 5 × 10^6^/kg was given to most patients, which might be due to the relatively lower disease burden and peak values of CAR-T cell expansion [6]. In other reports, CD19/22 dual targeting CAR-T cells [7, 8] and CD19/22 CAR-T cell cocktail therapy [9] did not increase the risk of severe CRS and neurotoxic effects. It was also reported that bispecific anti-CD20/CD19 CAR-T cells for relapsed B cell malignancies developed lower incidences of grade 3–4 CRS with 5%(1/22) and grade 3–4 neurotoxicity with 14%(3/22) [10]. Although the hematologic toxicity was the most common AE, it recovered quickly in the present study. These results suggested that the CD19/22 bispecific CAR-T cells for MRD-positive B-ALL patients had a higher safety.

Although the Cmax of CD19/CD22 CAR-T cell expansion was relatively lower, but it still had a good efficacy, which was consistent with the report by Park et al [11]. In their study, they found that a higher ratio of peak values of CAR-T cells expansion to tumor burden significantly correlated with EFS and OS. A higher dose of CAR-T cells for the majority of patients in our study might result in a higher ration of peak values of CAR-T cells expansion to tumor burden. In our study, CD19/CD22 bispecific CAR-T cell therapy resulted in an overall MRD response rate of 100% at day 28 assessment, indicating that the bispecific CAR-T cells had a rapid and efficient response rate for MRD-positive patients. The relapse incidence in our study was lower than that in other reports for relapsed/refractory B-ALL patients [7, 8], likely due to the enrolled patients with a lower disease burden, although three patients eventually experienced morphological relapse and 2 MRD relapse. For the 10 patients without subsequent transplantation after CAR-T in the first-line consolidation group, the 24-month RFS and OS were 77.8% and 88.9%; while for patients ≥45 years old, the 24-month RFS and OS were 77% and 88%. The result was similar to the report by Schultz LM et al. using Tisagenlecleucel for the patients with low disease burden of 1-year OS of 85% and EFS of 72%, respectively [12]. Allo-HSCT could eradicate MRD persistence in B-ALL patients, but 5-year OS was only 33% due to non-relapse mortality and relapse [13]. The immune reconstitution kinetics for CD3^+^, CD4^+^, and CD8^+^ were similar to other studies [14]. Till the end of the follow-up, 9 patients (4 Ph-negative and 5 Ph-positive) were with ongoing MRD negative remission and no history of allo-HSCT post CAR-T cell infusion. During to the relatively-shorter follow-up, we only propose that CD19/CD22 bispecific CAR-T therapy might be an optimal consolidation treatment modality for adult patients with MRD persistence after early consolidation chemotherapy, but it remains to be verified in further phase 2 clinical trials with larger sample size and a longer follow-up.

## Supplementary information

Supplementary materials

## Data Availability

The data that support the findings of the study are available on request from the corresponding author.
