# Correction: CD19/CD22 bispecific CAR-T cells for MRD-positive adult B cell acute lymphoblastic leukemia: a phase I clinical study

**DOI:** 10.1038/s41408-023-00825-7

**Published:** 2023-04-13

**Authors:** Jiahua Niu, Huiying Qiu, Fang Xiang, Lin Zhu, Jun Yang, Chongmei Huang, Kun Zhou, Yin Tong, Yu Cai, Baoxia Dong, Yuan Lu, Xuedong Sun, Liping Wan, Xueying Ding, Haopeng Wang, Xianmin Song

**Affiliations:** 1 Department of Hematology, Shanghai General Hospital, Shanghai Jiaotong University School of Medicine, Shanghai, China; 2 Engineering Technology Research Center of Cell Therapy and Clinical Translation, Shanghai Science and Technology Committee (STCSM), Shanghai, China; 3 Hrain Biotechnology, Shanghai, China; 4 School of Life Science and Technology, Shanghai Tech University, Shanghai, China

**Keywords:** Phase I trials, T cells, Acute lymphocytic leukaemia

Correction to: *Blood Cancer Journal* 10.1038/s41408-023-00813-x, published online 24 March 2023

In the fifth line of the second paragraph, the word of “negative” in “MRD-negative complete remission” should be “positive”.

The original article has been corrected.

