# Supplementary material for: CD19/CD22 bispecific CAR-T cells for MRD-positive adult B cell acute lymphoblastic leukemia: a phase I clinical study
Source: Blood Cancer J. 2023 Mar 24;13(1):44. doi: 10.1038/s41408-023-00813-x (PMC10039051; doi:10.1038/s41408-023-00813-x)
Supplement: Supplementary file 1 — Supplementary materials [file 41408_2023_813_MOESM1_ESM.docx]

## Supplementary Methods

## Study design and treatments

The study was an open, phase I clinical trial with a sample size of 16-18. The primary objective was to assess the safety of CD19/CD22 bispecific CAR-T cells for MRD positive B-ALL, and the secondary was to evaluate its efficacy. We performed traditional 1+1+3+3 dose escalation to determine the optimal single dose of CAR-T cells, which corresponded to four dose levels of 1× 10^6^ cells/kg, 2× 10^6^ cells/kg, 3× 10^6^ cells/kg, and 5× 10^6^ cells/kg, respectively. All enrolled patients underwent lymphodepletion with fludarabine 30 mg/m²/d for three days and cyclophosphamide 500 mg/m²/d for two days before CAR-T cell infusion. In addition, patients with Ph-positive ALL could receive TKI therapy 3 months after CAR-T cell infusion.

The clinical trial was conducted under the principles of the Declaration of Helsinki and approved by the Institutional Review Board of Shanghai General Hospital. All enrolled patients provided written informed consent. The clinical trial was registered at clinicaltrials.gov (NCT: 03919526).


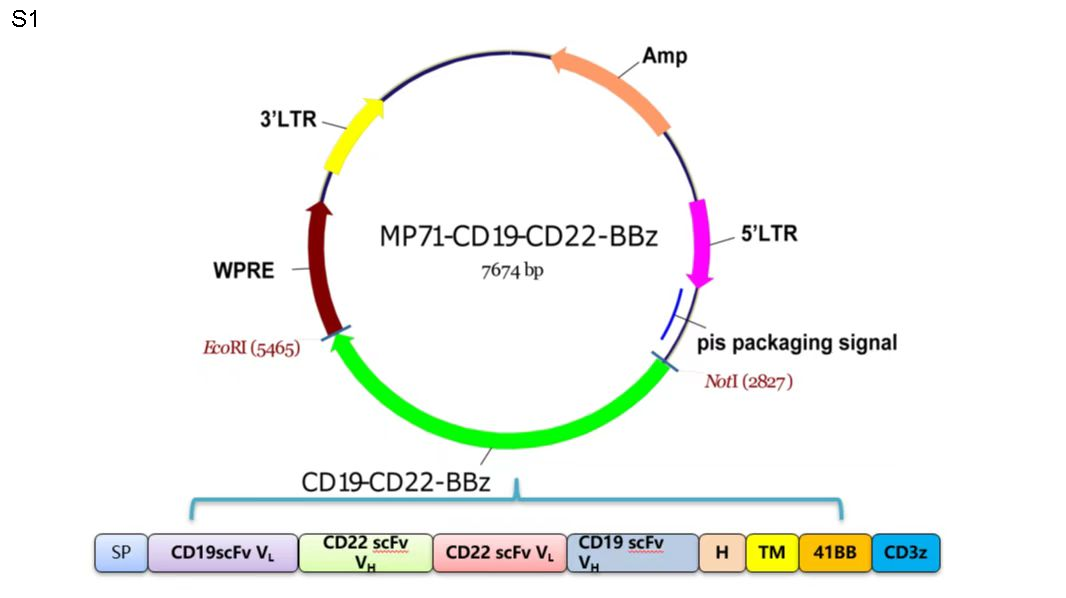


**Supplementary Figure 1. CAR 19-22 vector.** Anti-CD19/CD22 CAR consisted of anti-CD19 single-chain variable fragment (scFv) obtained from the murine monoclonal antibody FMC63, anti-CD22 scFv from a human antibody M971, a transmembrane region, and an intracellular fragment comprising the CD137 (4-1BB) costimulatory motif and the CD3-zeta (CD3ζ) signaling domain

## Safety evaluation

The safety of CD19/CD22 CAR-T cells was evaluated according to NCI Common Terminology Criteria for Adverse Events (CTCAE) v5.0. CRS was diagnosed and graded according to ASTCT grading system(1). Dose-limiting toxicities (DLTs) were evaluated according to CTCAE 5.0 as any toxicity of grade 3 or higher occurring within 28 days after CD19/CD22 CAR-T cell infusion possibly related to the treatment regimen. The criteria for DLTs included grade 4 or 5 CRS; or grade 3 CRS lasting for more than 7 days; organ toxicity of grade 3 or higher, or any unintended toxicity that requires termination of treatment. Neurotoxicity was graded according to CTCAE v5.0 and ASTCT Consensus Grading system. Neurologic symptoms were monitored twice daily after infusion, and were scored by ICE/ASTCT ICANS Consensus Grading system for adults. Management of ICANS is based on the severity of the symptoms and the concurrence of CRS. Grade 1 ICANS was treated with supportive care. If Grade1 ICANS was concurrence of CRS, tocilizumab will be used. For ≥Grade 2 ICANS, Dexamethasone 10mg intravenous every 6 h or methylprednisolone 1g intravenous will be used until symptoms improve.

## Efficacy evaluation

Response assessment was performed on day 14 and 28 (within 1 day) and thereafter every one month within the first year and every two months within the second year with bone marrow aspirate post-infusion. MRD negative was defined as less than 0·01% of leukemic cells by flow cytometry (FCM), and/or the absence of fusion gene by real-time quantitative PCR if available. CR, CR with incomplete hematological recovery(CRi), stable disease (SD), progressive disease (PD) and relapse were defined according to the 2021 Chinese Guideline for diagnosis and treatment of ALL (2). MRD relapse was evaluated based on NCCN criteria (3). Relapse-free survival (RFS) was defined as the time from infusion to the date of relapse or death from any cause. OS was defined from the time of CAR-T cell infusion to the date of death from any cause. All patients were followed up from the initiation of CAR-T cell infusion until relapse or death.

## Assessment of CAR-T cell expansion, persistence in vivo, serum cytokine levels and immune reconstitution

## Blood samples after infusion were collected on days -1, 0, +1, +3, +5, +7, +14, +28 and per month starting from the second month. The persistence of CAR-T cells was determined using flow cytometry (FCM) and quantitative PCR. Circulating CAR numbers were calculated on the basis of the measured absolute CD45^+^ T lymphocyte counts. Simultaneously, CAR DNA copies were evaluated as another method for determining the expansion and persistence. The levels of the cytokines including interleukin (IL)-6, C-reactive protein (CRP), IL-8，IL-10, TNF-α, and, IL-2 receptor in serum were measured at pre- and post-infusion with enzyme linked immunosorbent assay (ELISA) on day -1, 0, +1, +3, +5, +7, +14, +28 and per month starting from the second month. The lymphocyte subtype counts for CD3^+^, CD4^+^, CD8^+^, CD19^+^, CD20^+^and CD22^+^ cells were performed by multiflow cytometry at pre- and different time post-CAR-T cell infusion. Cell recovery was defined as ≥ lower limits of normal (LLN) counts of CD3^+^ cells (955/μL), CD4^+^ cells (550/μL), CD8^+^cells (320/μL), CD19^+^ cells (90/μL), CD20^+^ cells (90/μL), CD22^+^ cells (90/μL) according to the data of healthy population by our flow cytometry laboratory.

## Statistical analysis

Difference between two groups was analyzed by unpaired two-tailed Student's t-test. A chi-squared test was performed to estimate the response rates of patients with different doses of CAR-T cells and the frequency of fever between two groups. Pearson correlation coefficient was used for evaluating the correlation between different factors. The Kaplan-Meier approach was performed to estimate time-to-event analyses. All statistical analysis was performed using SPSS, and P values of less than 0.05 were considered significant.

**Structure of CD19/CD22 CAR**

CD19/CD22 CAR consisted of the signal peptide, anti-CD19 single-chain variable fragment (scFv) obtained from the murine monoclonal antibody FMC63 and anti-CD22 scFv from a human antibody M971(CD19 scFv V_L_-CD22 scFv V_H_ - CD22 scFvV_L_- CD19 scFv V_H_), the CD8α hinge and transmembrane domain, the 4-1BB costimulatory signaling domain, and the CD3 ζ cytoplasmic region. CD19/CD22 CAR was constructed based on a modified Moloney Murine Leukemia Virus (Mo-MLV)-based retrovirus vector, the clinical-grade retroviral producer cell lines for producing CAR vectors were generated with the use of the PG13 gibbon ape leukemia virus packaging cell line (CRL-10686, ATCC) (Supplementary Figure 1). CAR-T cells were generated by Hrain Biotechnology (Shanghai, China).Autologous T cells of patients were obtained from isolated peripheral blood mononuclear cells (PBMCs) and transduced with a retrovirus vector encoding the CD19/CD22 CAR.

**The manufacturing process and quality control assays**

CAR T cells were manufactured from cryopreserved apheresis product. In Brief, on day 1, cryopreserved apheresis product was thawed and followed by washing, CD3+ cells selection and activation by Dynabeads CD3/28 (Invitrogen) at ratio of 1:1 on day 2. On day 4, the activated cells were transduced with gamma retroviral vector in Retronectin-coated cell bags. Following transduction, Dynabeads were removed using CTS DynaMag (Invitrogen) and the transduced cells were further expanded. The medium used for culture was X-VIVO (Lonza) supplemented with 5% patient plasma, 1% GlutaMAX, 1% HEPES and 300 IU/mL IL-2. The culture was kept at 37℃ with 5% CO2. At the end of culture, cells were washed, formulated, frozen and kept in vapor phase of Liquid N2. All products must meet all releasing criteria before patient use.

**Supplementary Table 1 Inclusion and Exclusion criteria**

| Inclusion criteria | CD19 positive and / or CD22 positive B-cell acute lymphoblastic leukemia |
| --- | --- |
|  | 18 to 70 Years Old, Male and female |
|  | Expected survival > 12 weeks |
|  | ECOG score 0-2 |
|  | B-cell acute lymphoblastic leukemia was definitely diagnosed according WHO cretiria and met one of the following conditions:  a. For primary patients, CR was achieved after induction therapy, but MRD remained to be positive after at least 2 courses of consolidation therapy; For Ph-positive patients, at least one kind of TKIs was added into normative chemotherapy regimens;  b. For recurrent patients who achieved MRD-positive CR or CRi after salvage therapy or MRD relapse. |
|  | The venous access required for mononuclear cell collection can be established. |
|  | Liver, kidney and cardiopulmonary functions meet the following requirements:  a. Creatinine is in the normal range;  b. Left ventricular ejection fraction >50%;  c. Baseline oxygen saturation>92%;  d. Total bilirubin ≤ 2×ULN;  e. ALT and AST ≤ 2.5×ULN; |
|  | Able to understand and sign the Informed Consent Document |
|  | Malignant tumors other than acute lymphoblastic leukemia within 5 years prior to screening, not including adequately treated cervical carcinoma in situ, basal cell or squamous cell skin cancer, localized prostate cancer after radical resection, and ductal carcinoma in situ after radical resection |
|  | Subjects with positive HBsAg or HBcAb and peripheral blood HBV DNA titer detection ≥ 1 × 10^2^ copy number / L; HCV antibody positive and peripheral blood HCV RNA positive; HIV antibody positive; CMV DNA positive; syphilis positive |
|  | Any instability of systemic disease, including but not limited to unstable angina, cerebrovascular accident, or transient cerebral ischemic (within 6 months prior to screening), myocardial infarction (within 6 months prior to screening), congestive heart failure (New York heart association (NYHA) classification ≥ III), need drug therapy of severe arrhythmia, liver, kidney, or metabolic disease |
| Exclusion Criteria | Active or uncontrollable infection requiring systemic therapy within 14 days prior to enrollment |
|  | Pregnant or lactating woman, and female subject who plans to have a pregnancy within 1 year after cell transfusion, or male subject whose partner plans to have a pregnancy within 1 year after cell transfusion |
|  | Received CAR-T treatment or other gene therapies before enrollment |
|  | Patients with symptoms of central nervous system |
|  | Subjects who are receiving systemic steroid treatment and requiring long-term systemic steroid treatment during the treatment as determined by the investigator before screening (except inhalation or topical use); And subjects treated with systemic steroids (except inhalation or topical use) within 72h prior to cell infusion |
|  | The investigators consider other conditions unsuitable for enrollment |

**Supplementary Table 2 Patient baseline characteristics**

| **ID** | **Age** | **Sex** | **Cytogenetics at diagnosis** | **Mutated genes** | **Previous treatment** | **Cycle Number of chemo** | **Consolidation group** | **CAR-Tcell dose (10^6^/kg)** | **Response**  **(day 28)** | **RFS**  **(months)** | **OS**  **(months)** | **CRS**  **grade** | **Interval of CAR-T to HSCT** |
| --- | --- | --- | --- | --- | --- | --- | --- | --- | --- | --- | --- | --- | --- |
| 001 | 31 | F | Normal |  | chemo | 5 | First-line | 1 | CRi, MRD Neg | 2.5 | 2.5 | 0 | 2 |
| 002 | 57 | F | Normal |  | chemo | 3 | First-line | 2 | CR, MRD Neg | 6.5 | 7 | 0 |  |
| 003 | 70 | F | Normal |  | chemo | 4 | First-line | 3 | CR, MRD Neg | 33 | 33 | 0 |  |
| 004 | 23 | M | Normal | IKZF1 | chemo | 4 | First-line | 3 | CR, MRD Neg | 29 | 29 | 0 |  |
| 005 | 55 | M | Normal |  | chemo | 4 | First-line | 3 | CR, MRD Neg | 24 | 24 | 0 |  |
| 007 | 52 | F | Ph^+^ T315I |  | Chemo+TKI(pona) | 8 | Relapsed | 5 | CR, MRD Neg | 20.5 | 20.5 | 0 |  |
| 008 | 31 | F | Ph^+^ T315I | JAK3 | chemo+TKI(pona)+ Allo-PBSCT | 3 | Relapsed | 5 | CR, MRD Neg | 16.5 | 16.5 | 0 |  |
| 009 | 30 | F | Normal |  | chemo | 3 | First-line | 5 | CR, MRD Neg | 20 | 20 | 0 |  |
| 010 | 54 | M | Normal | TP53 | chemo | 3 | First-line | 5 | CR, MRD Neg | 17 | 17 | 1 | 11 |
| 011 | 25 | F | Ph^+^ |  | chemo+TKI(Dasa) | 4 | First-line | 5 | CR, MRD Neg | 15.5 | 15.5 | 2 |  |
| 012 | 51 | F | Ph^+^ T315I |  | chemo+TKI(Pona) | 5 | First-line | 5 | CR, MRD Neg | 15 | 15 | 1 |  |
| 013 | 37 | M | Complex karyotype | ETV6,  NF1 | chemo | 5 | First-line | 5 | CR, MRD Neg | 10 | 13.5 | 0 |  |
| 014 | 57 | M | Ph^+^ |  | chemo+TKI(Dasa)+ Allo-PBSCT | 4 | Relapsed | 5 | CR, MRD Neg | 3 | 14 | 0 |  |
| 015 | 61 | F | Ph^+^ | ATM,  CARD1,MTOR | chemo+TKI(Dasa) | 3 | Relapsed | 5 | CR, MRD Neg | 10.5 | 10.5 | 2 | 2.5 |
| 016 | 45 | M | Ph^+^_,_Complex karyotype | SMO,  SRP72,  CUX1 | chemo+TKI(Ima) | 3 | First-line | 5 | CR, MRD Neg | 6 | 6 | 0 |  |

Chemo: chemotherapy; Ph^+^: ph chromosome positive; Ima: imatinib; Dasa: dasatinib; Pona: ponatinib; CR: complete remission; MRD: measurable residual disease; neg: negative; RFS: relapse-free survival; OS: overall survival; CRS: cytokine release syndrome.

**Supplementary Table 3:** The transduction efficiency of CARs, calculated total number and the actual infused number of CAR-T cells for each patient

| Patient | Transduction efficiency | Dose (×10^6^ CAR-T cells/kg) | Weight (kg) | Calculated total CAR-T Cells (×10^6^) | Infused total CAR-T Cells (×10^6^) | Changes |
| --- | --- | --- | --- | --- | --- | --- |
| Patient1 | 37.7% | 1 | 60 | 60 | 64.5 | 7.50% |
| Patient2 | 70.3% | 2 | 55 | 110 | 111.5 | 1.36% |
| Patient3 | 73.1% | 3 | 70 | 210 | 208 | -0.95% |
| Patient4 | 68.9% | 3 | 75 | 225 | 218 | -3.11% |
| Patient5 | 65.9% | 3 | 70 | 210 | 220 | 4.76% |
| Patient6 | 54.6% | 5 | 61 | 305 | 346 | 13.44% |
| Patient7 | 50.0% | 5 | 53.5 | 267.5 | 275 | 2.80% |
| Patient8 | 62.8% | 5 | 40 | 200 | 202 | 1.00% |
| Patient9 | 61.9% | 5 | 74 | 370 | 403 | 8.92% |
| Patient10 | 64.6% | 5 | 59 | 295 | 272 | -7.80% |
| Patient11 | 57.2% | 5 | 61.2 | 306 | 345 | 12.75% |
| Patient12 | 54.1% | 5 | 92 | 460 | 468 | 1.74% |
| Patient13 | 55.1% | 5 | 62 | 310 | 307 | -0.97% |
| Patient14 | 39.4% | 5 | 48 | 240 | 236 | -1.67% |
| Patient15 | 20.3% | 5 | 63 | 315 | 312 | -0.95% |

The median transduction efficiency of CARs was 57.2% (range: 20.3%-73.1%). Patients were dosed at doses ranging from 1×10^6^ to 5×10^6^ CAR-positive viable T cells per kg weight (allowance of ± 15%).

**Supplementary Table 4.Adverse events of CD19/22 CAR-T cells within 28 days after infusion**

| **Adverse events** | Total | Grade 1 | Grade 2 | Grade 3 | Grade 4 |
| --- | --- | --- | --- | --- | --- |
| **Non-hematologic Adverse Events** | | | | | |
| **CRS** | 4 (26.7%) | 2 (13.3%) | 2 (13.3%) |  |  |
| **Neurologic toxic effect** | 1 (6.7%) | 1 (6.7%) |  |  |  |
| **Fever** | 7 (46.7%) | 5(33.3%) | 2 (13.3%) |  |  |
| **Productive cough** | 1 (6.7%) | 1 (6.7%) |  |  |  |
| **Malaise** | 2 (13.3%) | 2 (13.3%) |  |  |  |
| **Headache** | 1 (6.7%) |  | 1 (6.7%) |  |  |
| **Abdominal pain** | 1 (6.7%) | 1 (6.7%) |  |  |  |
| **Malabsorption** | 1 (6.7%) | 1 (6.7%) |  |  |  |
| **Hypocalcemia** | 4 (26.7%) | 4 (26.7%) |  |  |  |
| **Hyponatremia** | 6(40%) | 6(40%) |  |  |  |
| **Hypoglycemia** | 1 (6.7%) | 1 (6.7%) |  |  |  |
| **Hypoalbuminemia** | 14(93.3%) | 14 (93.3%) |  |  |  |
| **Hypercholesteremia** | 1 (6.7%) |  | 1 (6.7%) |  |  |
| **Hypoglycemia** | 6(40%) | 4 (26.7%) | 1 (6.7%) | 1 (6.7%) |  |
| **Hyperuricemia** | 1 (6.7%) | 1 (6.7%) |  |  |  |
| **Increased blood lactate dehydrogenase** | 7 (46.7%) | 6(40%) | 1 (6.7%) |  |  |
| **Hyperphosphatemia** | 1 (6.7%) | 1 (6.7%) |  |  |  |
| **Hypomagnesemia** | 3(20%) | 3(20%) |  |  |  |
| **Hypokalemia** | 1 (6.7%) | 1 (6.7%) |  |  |  |
| **Hypophosphatemia** | 2 (13.3%) | 2 (13.3%) |  |  |  |
| **Hypertriglyceridemia** | 13(86.7%) | 9(60%) | 3(20%) |  | 1 (6.7%) |
| **Hematologic Adverse Events** | | | | | |
| **Anemia** | 14(93.3%) | 6(40%) | 6(40%) | 2 (13.3%) |  |
| **Leukopenia** | 15(100%) |  | 4 (26.7%) | 6(40%) | 5(33.3%) |
| **Decreased Neutrophil count** | 15(100%) |  | 4 (26.7%) | 8(53.3%) | 3(20%) |
| **Decreased Lymphocyte count** | 15(100%) |  |  |  | 15(100%) |
| **Thrombocytopenia** | 3(20%) |  | 2 (13.3%) | 1 (6.7%) |  |

One patient (No.009) experienced a second grade 3 leukopenia at 45-55 days post infusion and recovered from granulocyte-colony stimulating factor.

**Supplementary Table 5 The cytokine levels after CAR-T cell infusion**

| **ID** | **IL-6 Tmax(d)** | **IL-6**  **Peak(pg/ml)** | **CRP Tmax(d)** | **CRP Peak(mg/L)** | **IL-15 Tmax(d)** | **IL-15**  **Peak(pg/ml)** | **TNF-α Tmax(d)** | **TNF-α Peak(pg/ml)** | **IFN-γ Tmax(d)** | **IFN-γ Peak(pg/ml)** | **GranzymeB Tmax(d)** | **GranzymeB Peak(pg/ml)** |
| --- | --- | --- | --- | --- | --- | --- | --- | --- | --- | --- | --- | --- |
| 001 | 3 | 6.6 | 3 | 73.21 | 1 | 12.16 | 3 | 56.5 | 14 | 19.2 | 20 | 500.1 |
| 002 | 7 | 22.98 | 10 | 92.77 | 1 | 18.93 | 28 | 8.3 | 7 | 6.6 | 10 | 66.57 |
| 003 | 5 | 15.13 | 5 | 31.81 | 1 | 14.88 | 29 | 4.2 | 7 | 4.9 | 7 | 429.1 |
| 004 | 1 | 30.03 | 3 | 29.84 | 1 | 39.08 | 10 | 6.64 | 7 | 4.63 | 7 | 24.38 |
| 005 | 7 | 204.7 | 3 | 226.9 | 1 | 23.12 | 14 | 5.1 | 21 | 4.9 | 5 | 122.9 |
| 007 | 3 | 17109 | 3 | 204.2 | 3 | 22.42 | 1 | 0.06 | 3 | 48.29 | 5 | 239.7 |
| 008 | 1 | 26 | 7 | 6.2 | 1 | 14.5 | 1 | 4.8 | 1 | 3.9 | 5 | 26.3 |
| 009 | 3 | 12.67 | 3 | 4.954 | 1 | 10.76 | 27 | 0.64 | 10 | 11.92 | 14 | 103 |
| 010 | 28 | 16.9 | 3 | 11.1 | 1 | 24 | 3 | 10.7 | 14 | 13.1 | 14 | 143.6 |
| 011 | 14 | 52 | 10 | 4 | 1 | 9.8 | 21 | 9.9 | 10 | 7.8 | 10 | 94.9 |
| 012 | 1 | 23.8 | 3 | 0.6 | 1 | 10.2 | 5 | 9.2 | 1 | 9.5 | 5 | 61.9 |
| 013 | 1 | 38.1 | 3 | 1 | 3 | 34.2 | 28 | 10.2 | 1 | 12.2 | 5 | 46.1 |
| 014 | 1 | 32 | 1 | 1 | 1 | 17.3 | 1 | 22.7 | 1 | 8.7 | 3 | 104.3 |
| 015 | 3 | 1799.26 | 7 | 111.32 | 7 | 27.83 | 7 | 13.91 | 7 | 33.04 | 7 | 229.95 |
| 016 | 1 | 37.16 | 1 | 148.63 | 1 | 74.31 | 21 | 12.75 | 3 | 17.47 | 1 | 60.53 |

Tmax: Time to Peak

**Supplementary history of Patient No.009**

Brain MRI showed multiple abnormal signals under and deep in the frontal parietal cortex with no enhancement; Electroencephalo-graph (EEG) was normal. The delayed neurotoxicity of CAR-T cells was suspected based on excluding CNS infiltration of leukemic cells, CNS infections, paraneoplastic syndrome and autoimmune encephalitis syndrome, etc. and having a history of a second CAR T-cell expansion on day 55 to 87 after infusion. The CNS symptoms were obviously alleviated a week after treatments with prednisone (15mg/d).

**Details of relapsed patients after CAR-T therapy**

Two out of the 11 patients in the first-line consolidation group (No. 002 and No. 013) experienced morphological relapse at 7 and 10 months post infusion with low CAR-T cells. Patient No. 002 refused salvage therapy and died of disease progression, and patient No. 013 was still alive through salvage chemotherapy. One patient (No.010) developed MRD relapse at 11 months post infusion without CD19 and CD22 expression loss or downregulation and regained an MRD negative CR by receiving an allo-HSCT. One patient (No. 001) underwent transplantation in MRD negative status and died from severe acute GvHD (aGvHD) at two months post-transplantation. Out of the 4 patients in the relapsed group, one (No.014) experienced morphological and CNS relapse at three months post-infusion, and regained an MRD negative through oral TKI (Olverembatinib) and intrathecal chemotherapy. One (No.015) developed MRD relapse and returned to MRD-negative CR after allo-HSCT. Except for one patient (No. 013) with a CD19 expression loss，all the other relapsed patients maintained the expression of CD19 and CD22. Two patients (No.6 and No.15) with CD19 single expression remain to be MRD negative until the end of the follow-up.

**PK/PD**

The median time to the peak CAR-T cell concentration (Cmax) was 10 days (range, 7–14) for the 15 patients. The median Cmax of CD19 and CD22 were 23924copies/μg DNA (range, 1853–121247) and 21010copies/μg DNA (range, 1962–117945), respectively (P=0.87), while the median AUC_0–28d_ of CD19 and CD22 were 205303 copies/μg of DNA × days (range, 21920–789505) and 200478 copies/μg of DNA × days (range, 19871–702943), respectively (P=0.744) (Supplementary Figure 6A). Cmax and AUC_0-28_of CAR T-cell expansion were higher in patients with sustained remission than that with relapse (P=0.048 for Cmax and P=0.018 for AUC_0–28d_, respectively)( Supplementary Figure 6B).

**Supplementary Table 6. The peripheral blood absolute counts (median±SD) of lymphocyte subtypes of patients at before and after CAR-T cells.**

|  |  | **Before lympho**  **depletion** | **After**  **Lympho**  **depletion** | **1month** | **2month** | **3month** | **4month** | **5month** | **6month** | **7month** | **8month** | **9month** | **10month** | **11month** | **12month** |
| --- | --- | --- | --- | --- | --- | --- | --- | --- | --- | --- | --- | --- | --- | --- | --- |
| CD3 | Median | 1003 | 29 | 577 | 669 | 894 | 531 | 984.5 | 783 | 1056 | 761 | 1006.5 | 738 | 1156.5 | 1041 |
|  | Min | 107 | 8 | 11 | 172 | 217 | 69 | 253 | 440 | 374 | 522 | 532 | 109 | 602 | 530 |
|  | Max | 1805 | 78 | 1891 | 2653 | 2436 | 1929 | 1794 | 2423 | 2591 | 2476 | 1569 | 2584 | 2243 | 1614 |
| CD4 | Median | 278.50 | 24.0000 | 115.00 | 138.0000 | 216.00 | 123.00 | 163.50 | 175.00 | 189.00 | 205.00 | 289.00 | 265.00 | 279.50 | 292.00 |
|  | Min | 0 | 5.00 | 5 | 12.00 | 65 | 21 | 91 | 121 | 125 | 124 | 140 | 45 | 225 | 157 |
|  | Max | 1403 | 49.00 | 352 | 983.00 | 418 | 483 | 481 | 498 | 448 | 579 | 411 | 598 | 621 | 510 |
| CD8 | Median | 400.00 | 8.0000 | 415.00 | 319.0000 | 630.00 | 351.00 | 443.00 | 536.50 | 650.00 | 563.00 | 555.00 | 524.00 | 814.00 | 534.00 |
|  | Min | 52 | 1.00 | 6 | 69.40 | 145 | 45 | 114 | 204 | 183 | 289 | 338 | 55 | 274 | 305 |
|  | Max | 803 | 24.00 | 1571 | 1447.00 | 1889 | 1424 | 1200 | 1841 | 2096 | 2007 | 1250 | 2077 | 1507 | 1070 |
| CD19 | Median | 19.00 | 1.0000 | 1.00 | 2.0000 | 7.00 | 23.00 | 85.00 | 79.50 | 90.50 | 75.00 | 52.00 | 104.00 | 178.50 | 169.00 |
|  | Min | 0 | 0.00 | 0 | 0.00 | 1 | 0 | 1 | 2 | 2 | 2 | 27 | 15 | 53 | 36 |
|  | Max | 240 | 4.00 | 5 | 76.00 | 37 | 99 | 248 | 2657 | 190 | 215 | 372 | 346 | 261 | 378 |
| CD20 | Median | 25.00 | 1.0000 | 1.00 | 2.0000 | 8.50 | 23.00 | 86.00 | 113.50 | 89.50 | 76.00 | 51.00 | 104.00 | 175.00 | 167.00 |
|  | Min | 0 | 0.00 | 0 | 0.00 | 1 | 0 | 1 | 2 | 2 | 2 | 26 | 14 | 99 | 34 |
|  | Max | 241 | 15.56 | 5 | 79.00 | 37 | 96 | 245 | 339 | 180 | 220 | 364 | 345 | 260 | 373 |
| CD22 | Median | 26.00 | 1.0000 | 2.00 | 2.0000 | 9.00 | 23.00 | 85.00 | 113.50 | 89.50 | 78.00 | 51.50 | 105.00 | 175.00 | 167.00 |
|  | Min | 0 | 0.00 | 0 | 0.00 | 1 | 0 | 1 | 2 | 2 | 2 | 26 | 14 | 98 | 34 |
|  | Max | 242 | 6.00 | 5 | 78.00 | 37 | 96 | 245 | 1866 | 180 | 219 | 363 | 347 | 250 | 372 |

The first time for the median CD3^+^, CD4^+^, CD8^+^, CD19^+^, CD20^+^, and CD22^+^ cells to reach LLNs after CAR-T cell infusion was at 5 months with 984.5 (253-1794)/µL；17 months with 626 (472-780) /µL；3 months with 630 (145-1889) /µL; 7 months with 90.5 (2-190) /µL；6 months with 113.5 (2-339) /µL；and 6 months with 113.5 (2-1866) /µL, respectively.


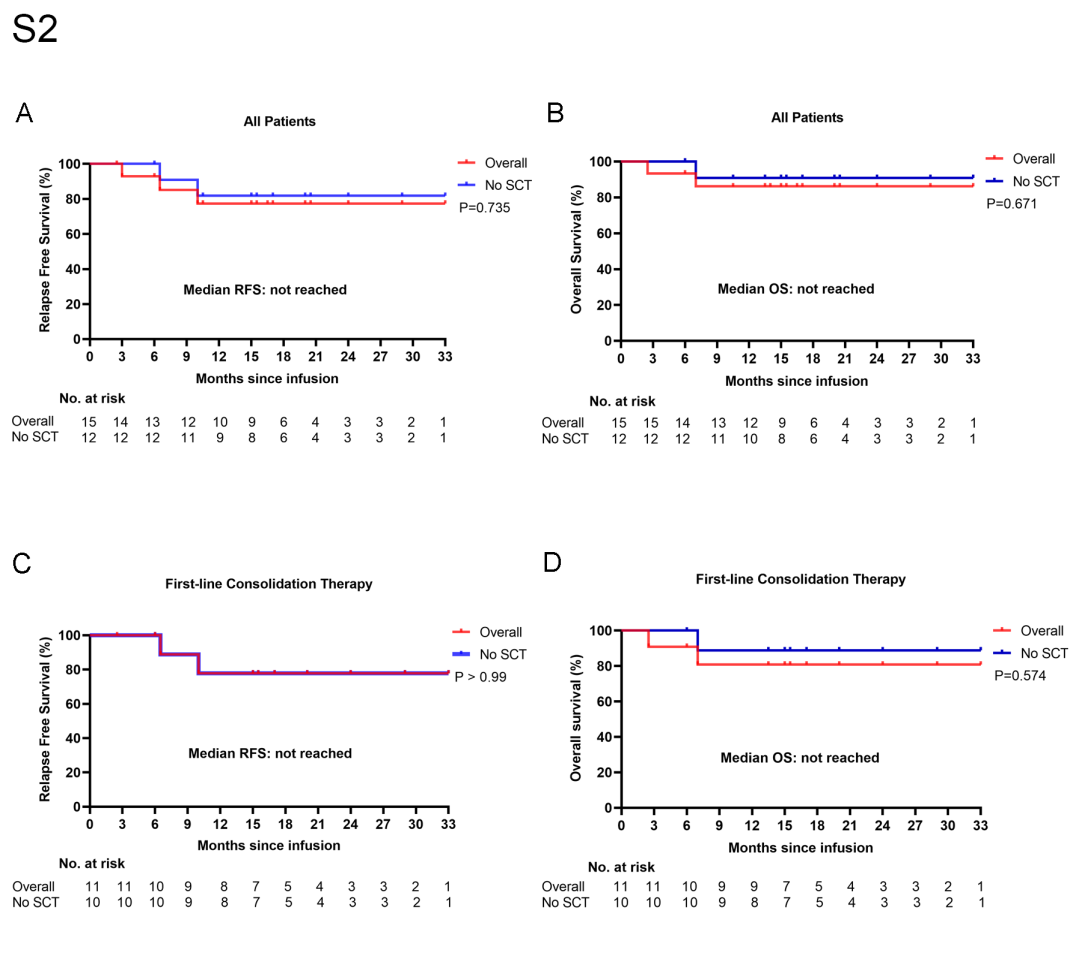


**Supplementary Figure 2. Survival of patients with MRD-positive B-ALL after CD19/CD22 bispecific CAR-T cells.** Kaplan–Meier analysis for RFS and OS for all patients and patients in first-line consolidation group after CAR-T cells, respectively. A and C: RFS; B and D: OS. No SCT: no transplantation after CAR-T cells.


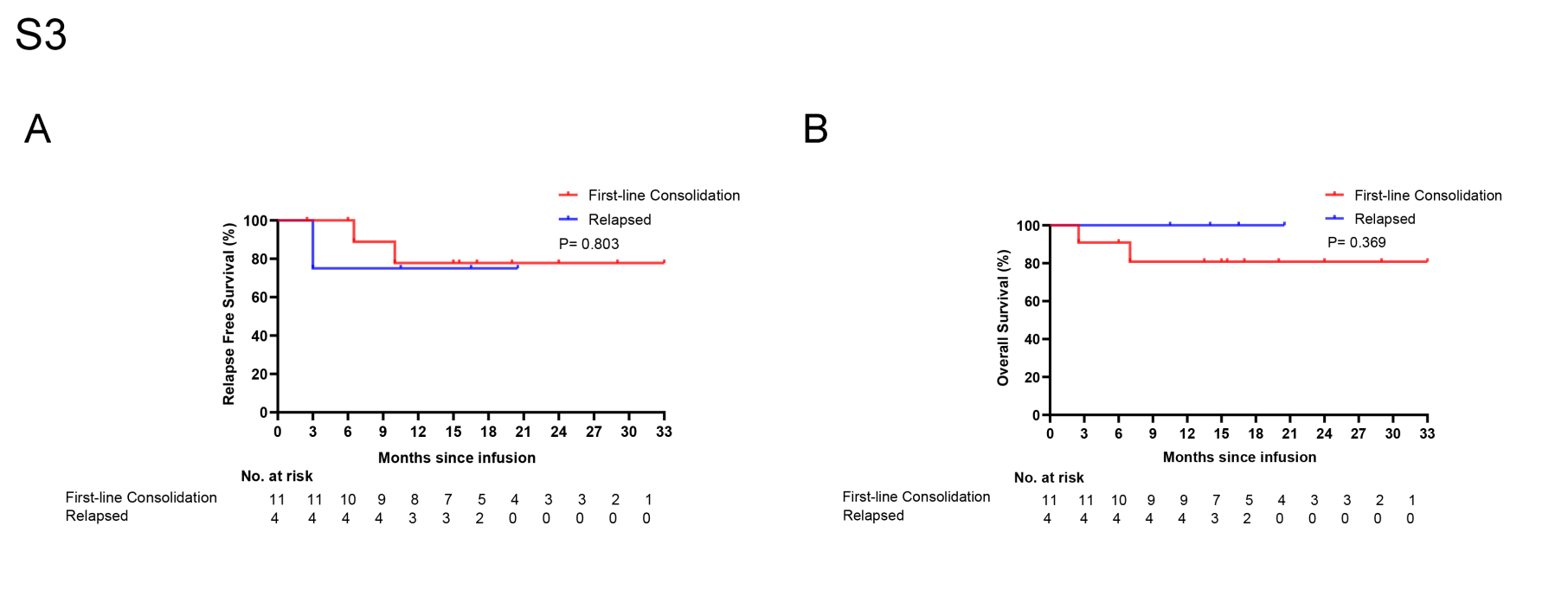


**Supplementary Figure 3. Survival of patients with MRD-positive B-ALL infused with CD19/CD22 bispecific CAR-T cells.** Kaplan–Meier analysis for RFS (A) and OS (B) of patients in first-line consolidation and relapsed group after CAR-T cells infusion.

**
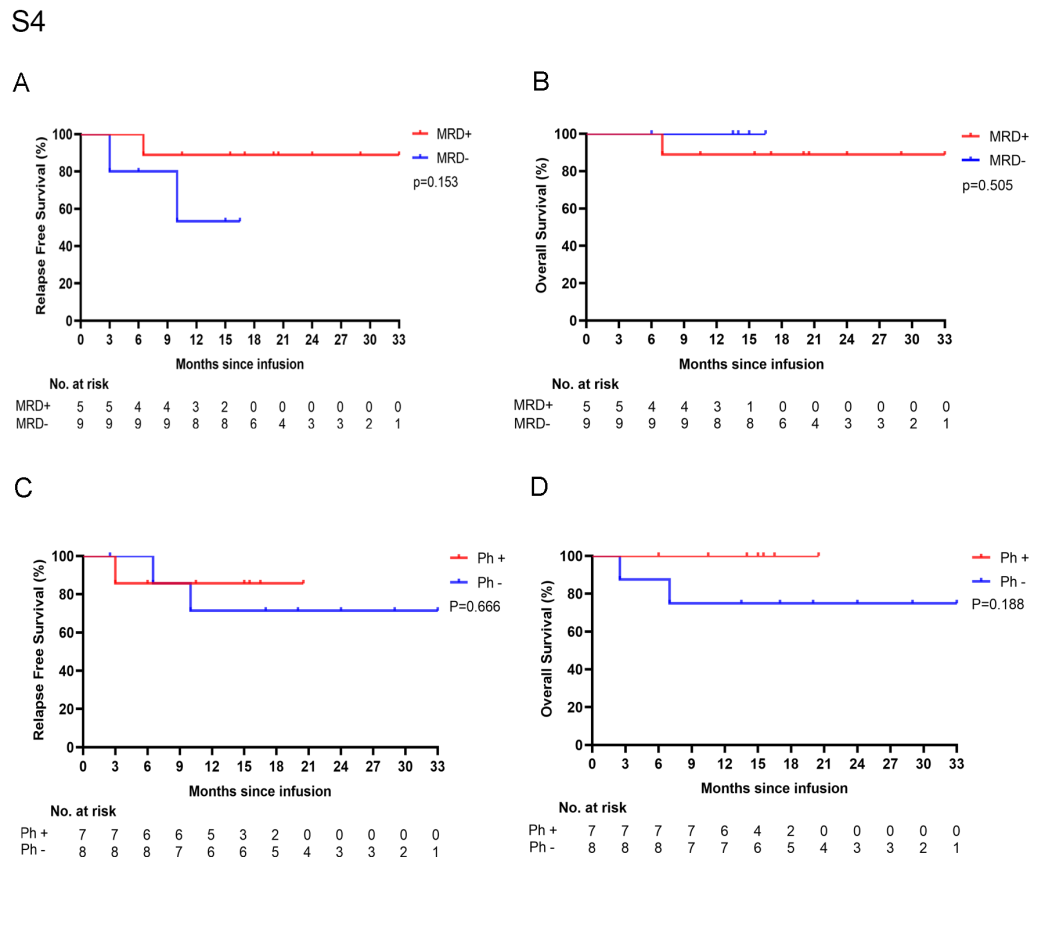
**

**Supplementary Figure 4. Survival of patients with MRD-positive B-ALL infused with CD19/CD22 bispecific CAR-T cells.** Kaplan–Meier analysis for RFS (A) and OS (B) according to MRD status before CAR-T cells infusion. Kaplan–Meier analysis for RFS (C) and OS (D) according to Ph status before CAR-T cells infusion.

**
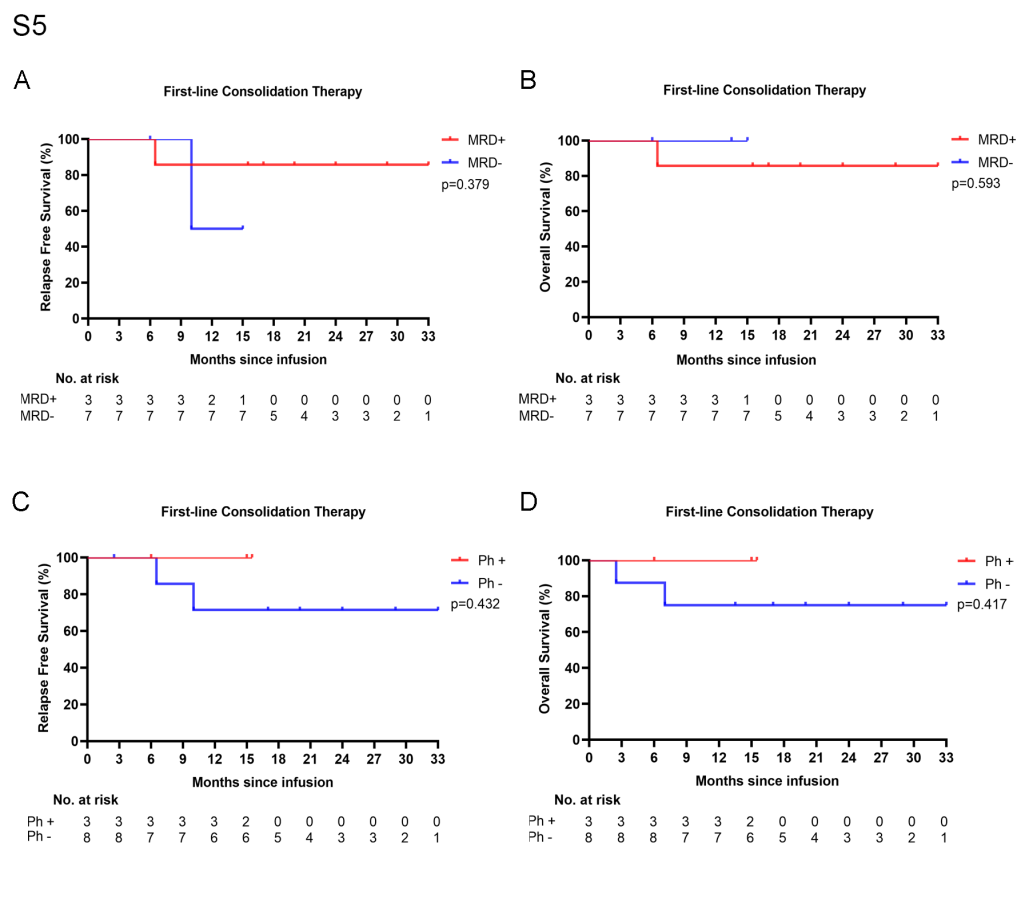
**

**Supplementary Figure 5. Survival of patients with MRD-positive B-ALL infused with** **CD19/CD22 bispecific CAR-T cells.** Kaplan–Meier analysis for RFS (A) and OS (B) of patients in first-line consolidation group according to MRD status before CAR-T cells infusion(P=0.379 for RFS, P=0.593 for OS). Kaplan–Meier analysis for RFS (C) and OS (D) of patients in first-line consolidation group according to Ph status before CAR-T cells infusion(P=0.432 for RFS, P=0.417 for OS).

**
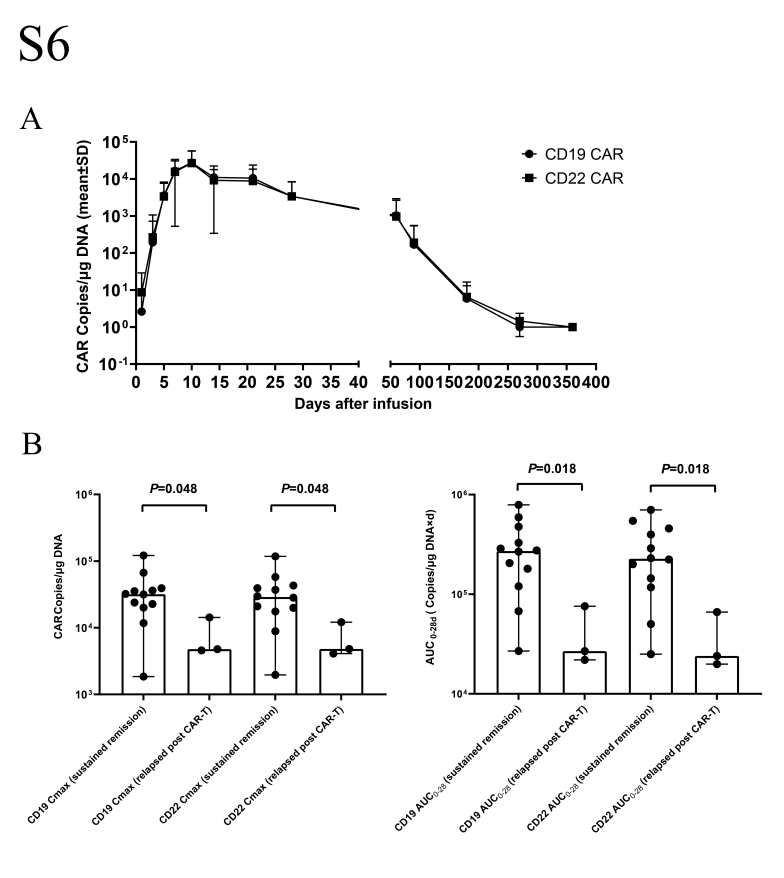
**

**Supplementary Figure 6. Expansion and persistence of CAR-T cells *in vivo*.**(A) The CAR-T cell expansion and persistence in peripheral blood (PB) after CAR-T cell infusion analyzed by fluorescence quantitative PCR. (B) and (C) The comparison of CAR T-cell expansion by peak (Cmax: B) and AUC_0-28_(C) in PB between patients with sustained remission and relapse after CAR-T cells.

**
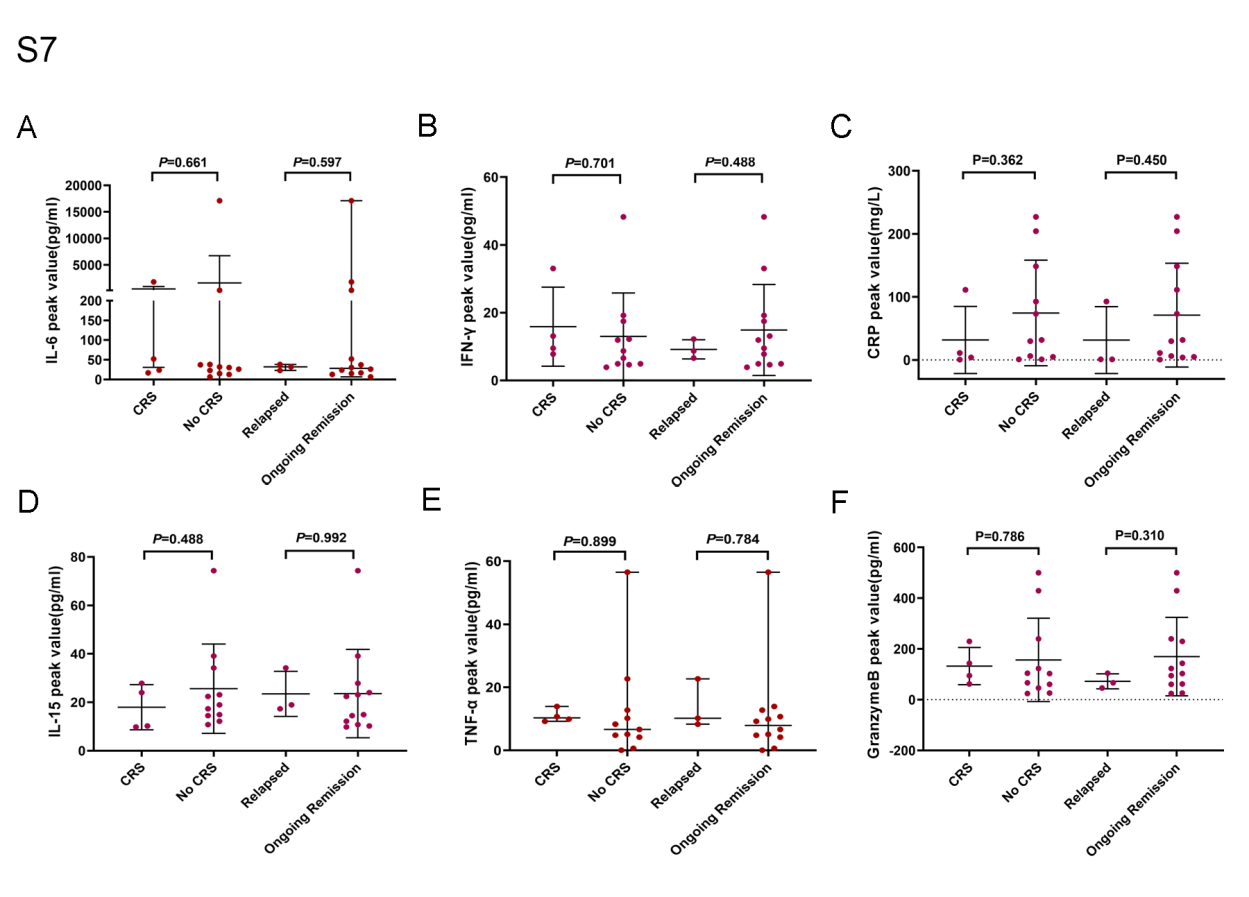
**

**Supplementary Figure 7.** Peak levels of IL-6(A), IFN-γ (B), CRP(C), IL-15(D), TNFα(E) and GranzymeB(F) within the first 30 days after infusion of CD19/CD22 bispecific CAR-T cells in patients between with and without CRS, and between with relapse and sustain remission.

**
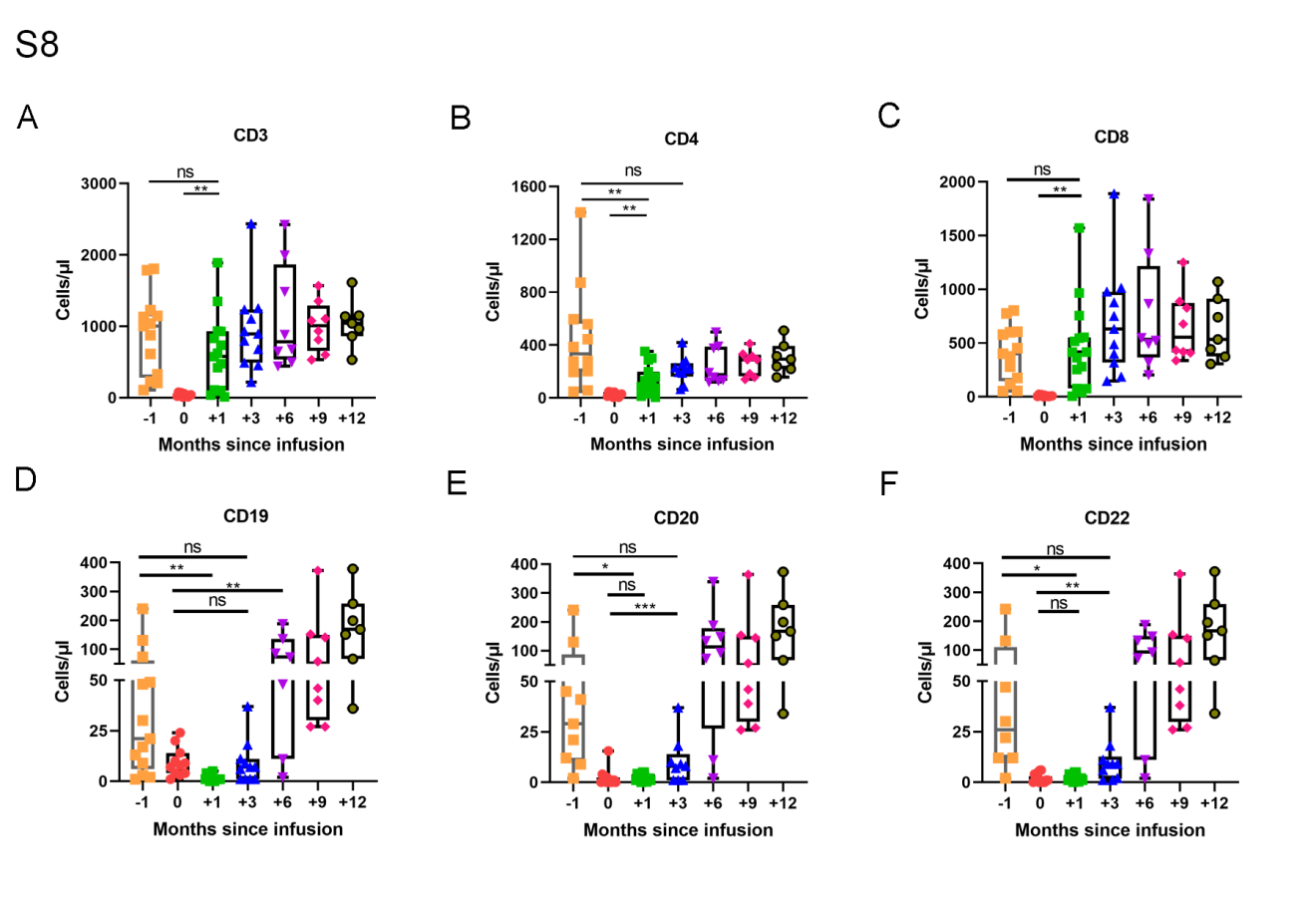
**

**Supplementary Figure 8. Immune reconstitution after CAR-T cells**. A: CD3^+^ cells; B: CD4^+^ cells; C: CD8^+^ cells; D: CD19^+^ cells; E: CD20^+^ cells; F: CD22^+^ cells. On the horizontal axis, -1 represents one month pre-infusion of CAR-T cells, 0 represents time at infusion of CAR-T cells and numbers such as +1 represent months post-CAR-T infusion.

1. Lee DW, Santomasso BD, Locke FL, Ghobadi A, Turtle CJ, Brudno JN, et al. ASTCT Consensus Grading for Cytokine Release Syndrome and Neurologic Toxicity Associated with Immune Effector Cells. Biol Blood Marrow Transplant. 2019;25(4):625-38.

2. Hematology Oncology Committee CA-CA, Leukemia, Lymphoma Group CSoHCMA. [Chinese guidelines for diagnosis and treatment of adult acute lymphoblastic leukemia (2021)]. Zhonghua Xue Ye Xue Za Zhi. 2021;42(9):705-16.

3. Brown PA, Wieduwilt M, Logan A, DeAngelo DJ, Wang ES, Fathi A, et al. Guidelines Insights: Acute Lymphoblastic Leukemia, Version 1.2019. J Natl Compr Canc Netw. 2019;17(5):414-23.
